# Chemical Modification of Polysaccharides: A Review of Synthetic Approaches, Biological Activity and the Structure–Activity Relationship

**DOI:** 10.3390/molecules28166073

**Published:** 2023-08-15

**Authors:** Tianbo Liu, Qianqian Ren, Shuang Wang, Jianing Gao, Congcong Shen, Shengyu Zhang, Yanhong Wang, Feng Guan

**Affiliations:** 1 School of Pharmacy, Heilongjiang University of Chinese Medicine, 24 Heping Road, Xiangfang District, Harbin 150040, China; liutianbo1997@163.com (T.L.); rqq2791989014@163.com (Q.R.); swhljucm@163.com (S.W.); jnghljucm@163.com (J.G.); scc16637647314@163.com (C.S.); tt13892638691@163.com (S.Z.); 2 Key Laboratory of Basic and Application Research of Beiyao, Ministry of Education, Heilongjiang University of Chinese Medicine, 24 Heping Road, Xiangfang District, Harbin 150040, China

**Keywords:** polysaccharide, chemical modification, biological activity

## Abstract

Natural polysaccharides are macromolecular substances with great potential owing to their wide biological activity and low toxicity. However, not all polysaccharides have significant pharmacodynamic activity; hence, appropriate chemical modification methods can be selected according to the unique structural characteristics of polysaccharides to assist in enhancing and promoting the presentation of their biological activities. This review summarizes research progress on modified polysaccharides, including common chemical modification methods, the change in biological activity following modification, and the factors affecting the biological activity of chemically modified polysaccharides. At the same time, the difficulties and challenges associated with the structural modification of natural polysaccharides are also outlined in this review. Thus, research on polysaccharide structure modification is critical for improving the development and utilization of sugar products.

## 1. Introduction

Polysaccharides are natural macromolecular carbohydrates made up of more than ten monosaccharides joined by different glycosidic bonds. They are one of the essential components of living organisms with an important role in life processes. Polysaccharides are widely found in plants, animals, bacteria and microorganisms [1]. Due to their good antioxidant [2], antitumor [3], immune regulation [4], antiviral [5], anticoagulant [6] and other biological activities, they have been the focus of scholars in the medical field. Since 1943, polysaccharides have been used as medicines to treat diseases [7] and, up until now, have gradually been developed into functional foods with the development of technology, which are well liked by people.

It has been confirmed that the biological activity of polysaccharides is closely related to their monosaccharide composition, category of glycosidic bond, spatial structure, molecular weight and branched chain structure [8]. However, not all natural polysaccharides have good biological activity, and some of them are not biologically active or their biological activity is relatively weak due to their special structure, decreasing their clinical therapeutic potential and efficacy. For example, due to their large molecular weight, some polysaccharides are difficult to be absorbed by the human body through the cell membrane, and thus cannot exert biological activity. It has been reported that appropriate structural modification can enhance the biological activity of polysaccharides for therapeutic purposes [9]. The chemical modification of polysaccharides can change their spatial structure, monosaccharide composition, monosaccharide molar ratio, molecular weight, as well as substituent type, position and number, to achieve the purpose of activity enhancement [10,11,12,13]. Therefore, the study of the chemical modification of polysaccharides has been the focus of polysaccharide analysis in recent years. At present, the commonly chemical modification methods include acetylation [14], sulfation [15], phosphorylation [16], selenization [17], carboxymethylation [18] and other chemical modification methods.

Numerous review articles have reported the methods of chemical modification for polysaccharides and changes in bioactivity [19,20,21,22]. However, there are few reports on the influencing factors of biological activity after chemical modification. The aim of this paper is to show the methods of chemical modification of polysaccharides, changes in bioactivity and factors affecting chemically modified polysaccharides, providing a reference for broadening the application of chemically modified polysaccharides in the field of pharmaceuticals and functional foods.

## 2. Methods for the Chemical Modification of Polysaccharides

Chemical modification is a method of modifying the structure of polysaccharides by introducing different kinds of reactive groups through chemical reagents to obtain derivatized polysaccharides. The chemical modification will cause the original hydroxyl group of the polysaccharide to be replaced by substituents. For example, the hydroxyl groups are replaced by groups such as acetyl, sulfate, phosphate, selenate and carboxymethyl groups. With the introduction of functional groups, information such as the molar ratio, spatial structure and molecular weight of monosaccharides also undergo corresponding changes in the polysaccharide. This has improved the problem of the low bioactivity of natural polysaccharides due to the shortcomings of physicochemical properties, such as high viscosity, poor water solubility and excessive molecular weight. It is vital for the research of conformational relationships of polysaccharides to select appropriate chemical modification methods, which can enhance or alter the biological activity of polysaccharides.

### 2.1. Acetylation Modification

**Figure 1:**
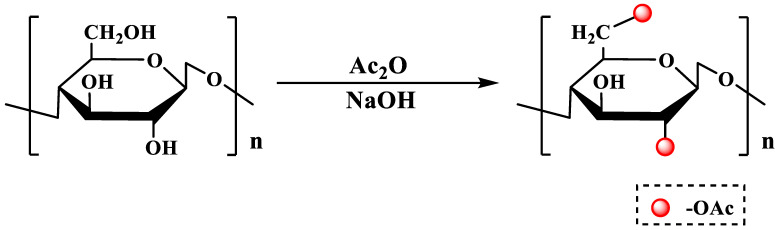
The acetylation modification of polysaccharides.

Acetylation modification is one of the important branched chain modification methods in polysaccharide chemical modification. Due to the introduction of an acetyl group, the hydroxyl groups have a nucleophilic substitution with acetic acid or acetic anhydride to generate acetate products, which can further change the spatial structure of the polysaccharides. This promotes the full expansion of the branched chains of the polysaccharide and further improves the solubility of the acetylated polysaccharide. This may be one of the reasons why acetylated modified polysaccharides have certain activities enhanced [14,19,23]. The polysaccharide acetylation reaction is shown in Figure 1.

Typical acetylation reagents include acetic anhydride or acetic acid. The acetylation reaction is performed by dissolving pre-prepared polysaccharides in organic solvents, such as formamide, dimethylformamide and DMSO, and then adding acetic anhydride or acetic acid reagents. As established, a suitable catalyzator is the key to a successful acetylation reaction. Common traditional acetylation catalysts mainly include pyridine and 4-dimethylaminopyridine (4-DMAP), as well as N-bromosuccinimide (NBS) [24,25]. It is worth noting that pyridine has a strong irritating odor and neurotoxicity, and although 4-DMAP has less toxicity than pyridine, it is more expensive and only suitable for laboratory-level research and development. These issues make them temporarily difficult to apply to large-scale production, resulting in limited application. However, reviewing the updated literature, many polysaccharide acetylation modification experiments do not add catalysts [26,27]. For example, polysaccharides can be directly dissolved in distilled water, and the pH value of the solution is adjusted to 9 with a NaOH reagent. Then, acetic anhydride is added, and NaOH is continued to maintain the pH value at 8–10 for a period of the reaction. Finally, HCl reagent is added to adjust the solution to being neutral, and acetylated polysaccharides are dialyzed to remove reaction by-products, and their concentration increased [28]. There are a lack of corresponding experiments for this phenomenon to clarify the relationship between the addition of catalysts and the degree of acetylation substitution.

### 2.2. Sulfation Modification

Sulfated polysaccharides refer to polysaccharides that contain sulfate groups on the sugar chain. It has been reported that various marine algal species contain sulfated polysaccharides [6]. In recent years, it has been found that sulfated polysaccharides have higher biological activity in terms of anticoagulation, antitumor and antioxidant activity compared to non-sulfated polysaccharides, which has attracted attention and made it one of the best choices for treating diseases [29,30]. However, in some species of marine polysaccharides, sulfated polysaccharides have better efficacy but lower content, which is difficult for use in large-scale clinical treatments. Therefore, it is urgent to synthesize sulfated polysaccharides using artificial chemical methods. As early as 1988, a Japanese scholar introduced sulfate groups to polysaccharides and found that the antiviral ability was enhanced, which established the basis for the artificial modification of sulfated polysaccharides [31]. At present, researchers’ interest in the synthesis of sulfated polysaccharides is mainly focused on methods such as concentrated sulfuric acid, chlorosulfate–pyridine and sulfur trioxide–pyridine methods [32]. However, after collecting articles from the past decade, it was found that the sulfamic acid method also seems to be helpful for the sulfation modification of polysaccharides [33,34]. In addition, the regional selective sulfation of polysaccharides is a very active research direction, ensuring the controllability and predictability of the introduction of sulfuric acid groups into polysaccharides and clarifying the structure–activity relationship [35].

#### 2.2.1. Sulfur Trioxide–Pyridine Method

**Figure 2:**
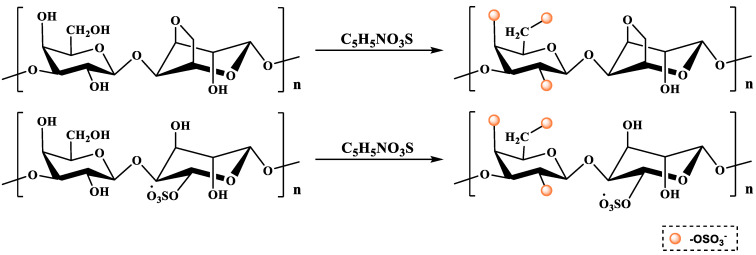
The sulfation modification of polysaccharide (i.e., the sulfur trioxide–pyridine method).

The sulfur trioxide–pyridine method is a milder sulfation modification method. First, the polysaccharide is completely dissolved in DMSO by stirring. Then, the esterification reagent prepared by adding a mixture of sulfur trioxide–pyridine dissolved in formamide is added, heated and stirred for a while. After the reaction is completed, the desired product is obtained via dialysis and freeze-drying (Figure 2) [36]. The product obtained using this method has a higher DS and is easy to operate and control. However, sulfur trioxide is relatively expensive and only suitable for small-scale production in the laboratory.

#### 2.2.2. Concentrated Sulfuric Acid Method

The concentrated sulfuric acid method is one of the classic sulfation methods. First, concentrated sulfuric acid and N-butanol are mixed in proportion, followed by adding ammonium sulfate and stirring in an ice-water bath. Then, the polysaccharide samples are added and stirred at a certain temperature, neutralized, precipitated with alcohol and freeze-dried to obtain the final products [37]. The concentrated sulfuric acid method has the advantages of producing a stable reaction and having low toxicity and low cost. However, it is less used nowadays mainly because concentrated sulfuric acid has strong acidity, which can easily cause polysaccharide carbonization and sugar-chain degradation.

#### 2.2.3. Chlorosulfonic Acid–Pyridine Method

The chlorosulfonic acid–pyridine method (CSA/Pyr) is currently the most widely used sulfation modification method, with the advantages of easy operation, high product yield and high DS. This method involves dissolving polysaccharides in formamide (or DMSO) and reacting with esterification reagents (chlorosulfonic acid–pyridine) under ice-water bath conditions for a period of time to obtain the products [38].

As a strongly oxidizing agent, chlorosulfonic acid is unstable and will react violently when exposed to water. In addition, it is flammable and highly toxic. The pyridine reagent also has a highly irritating odor. Nonetheless, this strategy is the best choice compared to the other two methods. Therefore, it is necessary to develop a less toxic and safer alternative to chlorosulfonic acid–pyridine sulfation.

#### 2.2.4. Sulfamic Acid Method

Compared to the preceding methods of sulfation, sulfamic acid seems to have received less attention. First, the polysaccharide sample is crushed and placed in a beaker, and then sulfamic acid and N, N-dimethylformamide are added. The mixture is then reacted in a water bath at 100 °C for 5 h. After the reaction is completed, the product is placed for cooling, neutralized with NaOH, dialyzed and freeze-dried to obtain the target product [33]. In addition, some scholars have used the amino sulfonic acid method to optimize the Box–Behnken process of guar gum galactomannan. The optimal process occurs upon adding 34 mmol of sulfamic acid to 1 g guar gum galactomannan at 85 °C for 2.6 h [34]. The results indicate that the reaction conditions for preparing the product using the amino sulfonic acid method are mild and that the reagent toxicity is low. However, its drawbacks are still obvious. The disadvantage of the sulfation reaction using the sulfamic acid method is that the reaction activity is low. Therefore, catalysts such as pyridine, urea and acetamide are usually required for catalytic reactions. At the same time, it is easily accompanied by the side reactions of carbamate. In recent years, the development of the amino sulfonic acid method has not been sufficient, so the sample size is smaller compared to the previous three methods.

### 2.3. Phosphorylation Modification

In nature, phosphorylated polysaccharides are mostly found in animal and plant species, with phosphate esters as the main form. However, because of their low content, limited variety and difficulty in extraction and isolation, they are generally synthesized artificially using chemical modification methods [39]. Under suitable conditions, polysaccharides can react with phosphorylation reagents so that the side chains and phosphorylation groups exist in a covalent manner. In addition, common contemporary phosphorylation modification methods mainly include phosphoric acid and its anhydride method, phosphorus oxychloride method, phosphate salt method and phosphorus pentoxide method.

#### 2.3.1. Acid and Anhydride Methods

**Figure 3:**
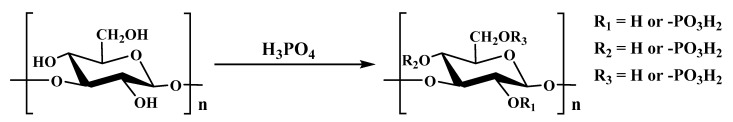
The phosphorylation modification of polysaccharides (i.e., the acid and anhydride method).

The acid and acid anhydride methods were applied earlier in the modification of polysaccharide phosphorylation, whose reaction process is relatively simple. Briefly, polysaccharide powder is dissolved in a mixture of urea and DMSO. Then, phosphoric acid is added, and it is reacted at 100 °C for 6 h. In the end, the product is dialyzed and lyophilized (Figure 3) [40]. This method has the advantages of simple operational steps, universal instrument applicability and low cost. However, due to the intense phosphoric acid reaction process, which generates a large amount of heat, it can lead to the degradation of polysaccharides and lower yield of the target product, so it currently has few applications.

#### 2.3.2. Phosphorus Oxychloride Method

The phosphorus oxychloride method, also known as the phosphoryl chloride method, is a method for synthesizing highly substituted phosphorylated products. Its advantages include a rapid reaction time, simple operation and high DS [41]. First, the polysaccharide powder is dissolved in DMF, and a mixture of phosphorus oxychloride (POCl_3_) with pyridine is slowly added under the condition of an ice-water bath to react at a specific temperature and time. After that, phosphorylated polysaccharides are obtained through alcohol precipitation, centrifugation and freeze-drying [16]. Although the phosphorus oxychloride method is widely used, it also has certain drawbacks, such as a relatively violent reaction and highly toxic byproducts, and the reaction will also be accompanied by the production of irritating gases.

#### 2.3.3. Phosphate Method

At present, the phosphate method is a commonly used method for phosphorylation modification. Compared with other methods, the phosphate method has the superiority of not easily degrading polysaccharides. However, the disadvantages are also obvious, such as the low reaction activity of polysaccharides when phosphorylated, which results in the DS and yield of products being relatively low. Commonly used phosphates are sodium tripolyphosphate (STPP) and sodium trimetaphosphate (STMP) in phosphorylation modification [42]. Specifically, polysaccharide powder is dissolved in distilled water, and STPP and STMP are added in a 6:1 ratio and according to optimal phosphorylation conditions. The reaction lasts for 6 h at 80 °C, and the target product is obtained via neutralization, dialysis and freeze-drying at the end of the reaction [43].

#### 2.3.4. Phosphorus Pentoxide Method

Chitosan (CSSA) alkylated with stearic acid is mixed with pre-cooled methanesulfonic acid. After process optimization, the best ratio is selected to add to it four times the proportion of phosphorus pentoxide (P_2_O_5_) to that of CSSA. Then, the mixture is stirred at 0–5 °C for 1 h. Finally, the target product is obtained via ether precipitation, centrifugation, neutralization or dialysis [44]. However, P_2_O_5_ has a strong acidity which can easily cause the degradation of polysaccharides during the reaction process and also lead to a low DS of the products. Therefore, its current application is limited.

### 2.4. Selenization Modification

Selenided polysaccharides are formed by the combination of polysaccharides and inorganic selenium through covalent bonds. Numerous studies have shown that selenided polysaccharides have stronger biological activity and better absorption properties than original polysaccharides and inorganic selenium [45,46,47]. Selenided polysaccharides exist only in trace amounts in natural microorganisms and plants. However, due to their low content and limited variety, selenium polysaccharides’ development and utilization are limited. Until now, the synthesis of selenium polysaccharides was often carried out through artificial synthesis, with the aim of increasing the bioavailability of selenium polysaccharides and thus expanding their application scope. There are many common types of chemical modification methods for selenium polysaccharides at present, mainly divided into two categories, i.e., the selenate method and the selenium oxychloride method.

#### 2.4.1. Selenate Method

**Figure 4:**
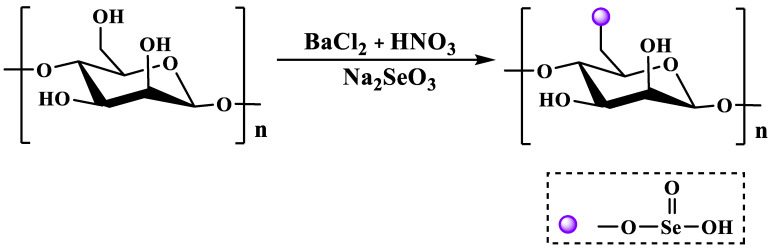
The selenization modification of polysaccharides (i.e., the NA-SS method).

The method of using selenite and its salts as selenide reagents is called the selenate method, and common selenite salts include sodium selenite. Therefore, according to different reaction systems, it can be divided into the nitric acid sodium selenite method (NA-SS) [46], the glacial acetic acid sodium selenite method (GA-SS) [48], the nitric acid selenite method (NA-SA) [49] and the glacial acetic acid selenite method (GA-SA) [48]. BaCl_2_ is often used as a catalyst in the GA-SA method due to its strong coordination with hydroxyl groups. Among them, the NA-SS method is currently the most widely used selenization method due to its simple operation and high degree of selenization. Briefly, the specific operation process consists of dissolving polysaccharide powder in nitric acid at 25 °C, followed by the addition of Na_2_SeO_3_ and maintaining the mixture in an oil bath at 70 °C for 10 h. After the reaction, the selenized polysaccharide is obtained via neutralization, dialysis and freeze-drying [50]. The NA-SS method modifies the polysaccharides as shown in Figure 4.

#### 2.4.2. Other Selenization Methods

In addition to the selenate method, there is also the selenium oxychloride method (SeOCl_2_) for the chemical modification of polysaccharides [51]. However, the application of this reaction is currently limited because SeOCl_2_ is unstable and easily decomposes compared with the selenate method and is accompanied by irritating toxic gases. Except for the chemical synthesis of selenated polysaccharides, selenium polysaccharide can also be artificially synthesized via plant [52] and microbial transformations [53].

### 2.5. Other Methods for the Chemical Modification of Polysaccharides

In addition to the common chemical modification methods mentioned above, other methods for the chemical modification of polysaccharides consist of carboxymethylating [54], benzoylating [55], alkylating [56], hydroxypropylating [57], etc. As is well established, the pharmacological activity of most compounds depends on their structure. Chemical modifications of polysaccharides have become one of the main focuses in the field of polysaccharide research. They are used to modify the internal structure of polysaccharides, thereby improving biological activity and clarifying their conformational relationships. The advantages and disadvantages of chemical modification methods for polysaccharides are presented in Table 1.

## 3. Biological Activities of Chemically Modified Polysaccharides

### 3.1. Antioxidant Capacity

Free radicals are metabolic products in the body, and active oxygen radicals such as superoxide anion (O^2−^) and hydroxyl radical (·OH) and –NO groups are produced during normal life activities. An appropriate amount of reactive oxygen radicals will participate during normal life activities, but excessive accumulation of free radicals will lead to the occurrence of lipid peroxidation [58,59,60]. It can lead to a series of diseases such as chronic kidney disease [61], cancer [62] and cardiovascular disease [63]. Therefore, it is of great significance to search for natural antioxidants with low toxicity. In recent years, relevant studies have shown that chemically modified polysaccharides have the advantages of good antioxidant activity, low toxicity and being extensive medicinal resources, which have become a research hotspot in the scientific community.

**Figure 5:**
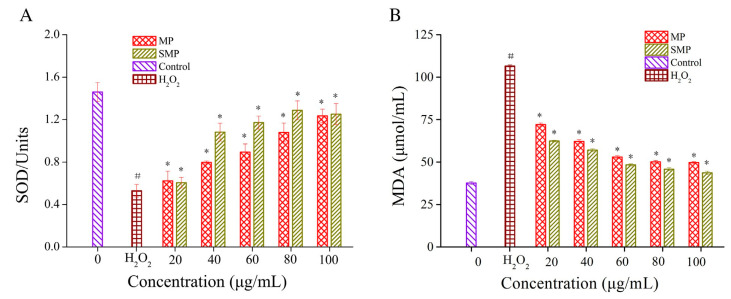
Effects of polysaccharides (MP) from Mesona chinensis Benth and SMP on the activities of SOD (**A**) and MDA content (**B**) in RAW264.7 cells. ^#^ *p* < 0.05 compared with normal group, * *p* < 0.05 compared with H_2_O_2_ group alone. This figure was adapted from Huang et al. [66].

The DPPH and superoxide anion systems can effectively evaluate the antioxidant activity of substances under in vitro antioxidant conditions. The latest research results show that the ability of polysaccharides (PYPs) from *Porphyra yezoensis* to scavenge DPPH and hydroxyl radicals increases with the introduction of sulfate groups. The reason may be that with the increase in sulfuric acid groups, the hydrogen atoms on the end-group carbon become active, increasing their own nucleophilic properties and thus enhancing the antioxidant capacity [33]. Due to the introduction of carboxymethyl groups, carboxymethylated polysaccharides (CRNPs) from blackcurrant fruits have stronger anti-lipid peroxidation capacity and free radical-scavenging abilities than original polysaccharides [64]. In addition, chemically modified polysaccharides also have outstanding antioxidant effects in vivo. With the introduction of selenium, selenified Chinese angelica polysaccharides (CAPs) are able to increase superoxide dismutase (SOD) and total antioxidant capacity (T-AOC) activities, significantly reducing the content of malondialdehyde (MDA) and reactive oxygen species (ROS) in liver tissues, and they have a significant antioxidant effect [65]. Sulfated polysaccharides (SMP) from *Mesona chinensis* Benth have good free radical-scavenging performance, which can reduce the content of MDA and improve the activity of SOD. Therefore, they exhibit excellent results in the ability to protect cells from oxidative stress (Figure 5) [66]. The biological activity and related mechanisms of chemically modified polysaccharides are shown in Table 2.

### 3.2. Antitumor Activity

Tumor disease is one of the most life-threatening and incurable diseases, which has attracted wide attention from researchers [67]. Most existing chemical drugs for cancer treatment also cause very serious damage to normal cells. Natural plant polysaccharides and their derivatives have a greater future prospect due to their good antitumor activity and low toxic side effects [68,69].

**Figure 6:**
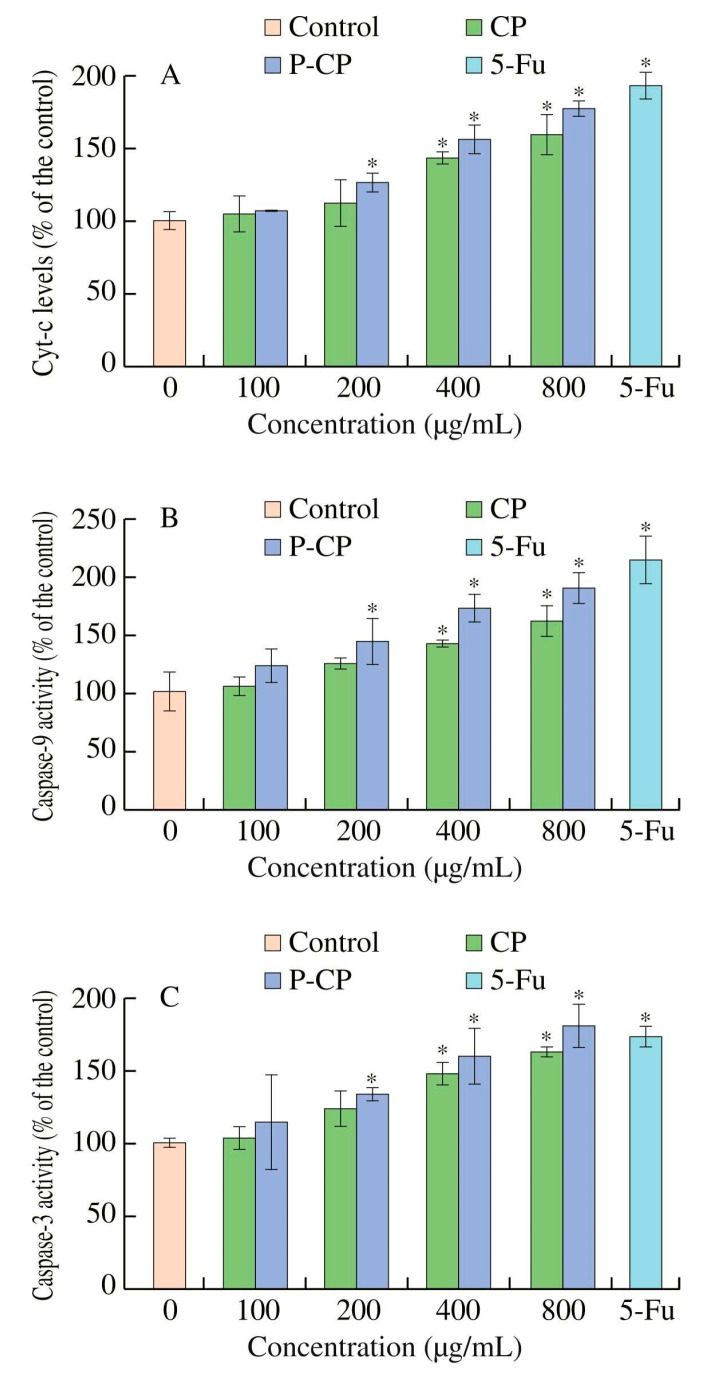
Polysaccharides (CP) from *Cyclocarya paliurus* and phosphorylated polysaccharides (P-CP) from *Cyclocarya paliurus* induced the activation of Cyt-c (**A**), caspase-9 (**B**) and caspase-3 (**C**) in CT-26 cells. * *p* < 0.05, compared with the untreated group. This figure was adapted from Xie et al. [72].

In vitro cellular experiments have shown that selenized polysaccharide (EJP90-1-Se) from *Eriobotrya japonica* with the introduction of the selenium element significantly inhibited cancer cell proliferation by inducing apoptosis. Further verification using a zebrafish model showed that EJP90-1–Se had a stronger inhibition of HepG2 proliferation and angiogenesis than polysaccharide (EJP90-1) from *E. japonica* [46]. Previous in vitro experiments have shown that sulfated polysaccharides (ASPs) from *Artemisia sphaerocephala* can significantly inhibit HeLa cells and HepG2. Meanwhile, according to the cell cycle, ASPs can block H22 cells in the S phase, thereby achieving antitumor effects through cell apoptosis. With other antitumor drugs, no direct cytotoxic effect on mouse fibroblast L929 was observed, while ASPs were antitumor [70]. It has been reported that sulfated polysaccharides may also achieve antitumor effects by reducing tumor microvascular density (MVD) and inhibiting the expression of vascular endothelial factor [71]. According to the latest research, phosphorylated polysaccharides are effectively modified to enhance antitumor activity. The mechanism may be to increase the activity of Cyt-c, Caspase-3 and Caspase-9, to induce cell apoptosis and also to arrest the cell cycle in the S phase (Figure 6) [72]. In addition, it has also been shown that sulfated polysaccharides can stimulate the activity of lymphocytes, increase macrophage phagocytosis and improve the production of large amounts of cytokines in macrophages. This can activate the immune response, thus possessing good proliferative activity against HONE1 cells [37]. The reason for enhanced immunity is that sulfuric acid groups increase contact with immune cell receptors by combining oxygen and electrostatic attraction, enhance the immune response and then inhibit the proliferation of tumor cells.

### 3.3. Antiviral Activity

The antiviral mechanism of chemically modified polysaccharides mainly operates through the inhibition of virus replication, enhancement of immune function and prevention of virus adsorption and invasion. Sulfated polysaccharide (SP) from *Sargassum ilicifolium* can prevent the adsorption and entry of viruses, thus achieving an antiviral effect. At the same time, SP is much less toxic compared to other antiviral drugs [73]. Some scholars have successfully synthesized phosphorylated polysaccharides (pCPPSs) from *Codonopsis pilosula*, and in vitro and in vivo experiments have shown that pCPPS was able to block the formation of autophagosomes, thereby inhibiting the replication of the duck hepatitis A virus (DHAV) genome and achieving antiviral effects [74]. In addition, enhancement of the body’s immune response is also one of the antiviral mechanisms of chemically modified polysaccharides. Sulfated polysaccharides (sCVPSs) from *Chuanmingshen violaceum* can significantly reduce the virus titer in the thymus, spleen, brain and lungs of diseased chickens. The detection of serum interferon α and γ concentrations allowed for the conclusion that the antiviral effect of sCVPS was due to immune enhancement [75].

### 3.4. Immunomodulatory Activity

Immune function is a defense system for the body to maintain the relative stability of the internal environment and remove invading body antigens. Plant polysaccharides can perform immunomodulatory functions in the following method: regulating the secretion of cytokines, regulating signaling pathways such as mitogen-activated protein kinase (MAPK) and nuclear factor-κB (NF-κB), regulating intestinal flora and ameliorating organ failure.

**Figure 7:**
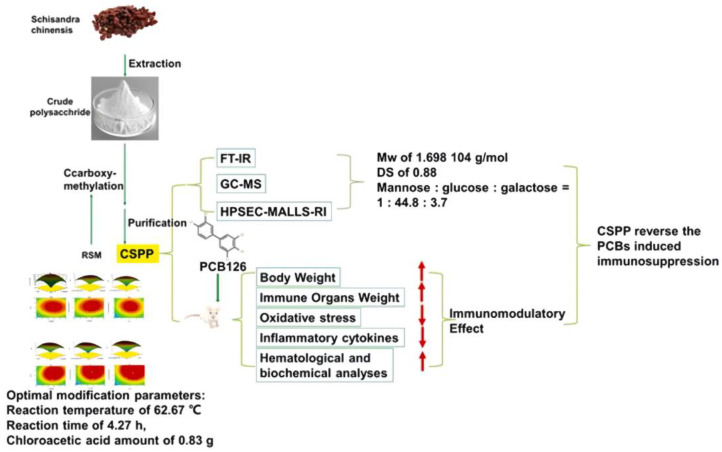
Preparation and characterization of carboxymethylated polysaccharides and their intervention in the immunotoxicity of polychlorinated biphenyls. This figure was adapted from Zhao et al. [80].

In the short term, it has been elucidated by RNA-seq that sulfated polysaccharide (S-CYP) from Chinese yam can achieve immune regulation by regulating the MAPK signaling pathway. At the same time, it can synergistically enhance immune function by increasing the secretion of cytokines [76]. In addition, it has been shown that sulfated polysaccharides also have the ability to stimulate an increase in NF-κBp65 protein and simultaneously block the action of TLR2/4, thereby passing through the NF-κB signaling pathway for immune regulation [77]. The most common ways of immune regulation modalities for chemically modified polysaccharides include the stimulation of macrophage activity and enhancement of cytokine secretion [27,78]. A small amount of research has shown that sulfated polysaccharides can enhance immune function by promoting the repair of intestinal mechanical barriers and regulating intestinal microflora. Compared to unmodified polysaccharides, sulfated modified polysaccharides have significantly increased immune regulatory activity [79]. Similarly, carboxymethylated polysaccharide (CSP) from *Schisandra chinensis* inhibited the thymic and splenic atrophy induced by dioxins such as 3, 3′, 4, 4′, 5-pentachlorobiphenyl (PCB 126). Moreover, it showed higher immunomodulatory activity compared to unmodified polysaccharides (Figure 7) [80].

### 3.5. Anti-Inflammatory Activity

The development of inflammation is an extremely complex process, and inflammation is usually a natural protective response of the body. However, long-term inflammation or inflammation that attacks oneself is harmful and can lead to a series of diseases in the body [81]. Therefore, the development of safe and effective anti-inflammatory drugs currently has a broad clinical application value. Natural macromolecular polysaccharides have received widespread attention from scientists because of their significant anti-inflammatory effects and high safety profile.

It has been reported that phosphorylated polysaccharide (PPN) from *Pholiota nameko* has anti-inflammatory effects on lipopolysaccharide (LPS)-induced RAW 264.7 cells through inhibition of the PI3K/AKT/mTOR pathway. Furthermore, the anti-inflammatory effect is consistently superior to that of polysaccharides without phosphorylation modification at the same concentration [82]. On the other hand, some studies have shown that acetylated polysaccharides also have good anti-inflammatory activity, for which the mechanism is to enhance anti-inflammatory activity through the NF-κB and p38/MAPK signaling pathways, as well as a strong ability to inhibit nitric oxide (NO) production [26]. Not only do artificially synthesized modified polysaccharides have good anti-inflammatory activity, but naturally occurring sulfated polysaccharides also have the same effect. Sulfated polysaccharide (CFCE-PS) from *Codium fragile* was able to dose-dependently reduce the levels of inflammatory factors in LPS-induced RAW264.7 cells, including NO, TNF-α, IL-1β, IL-6, etc. [83].

**Table 2:** Structure, biological activity and mechanism of chemically modified polysaccharides.

Bioactivity	Polysaccharide Sources	Monosaccharide Composition	Monosaccharide Composition of Modified Polysaccharide	Structures	Chemical Modification Methods	Mechanism	Ref.
Antioxidant	*Porphyra haitanensis*	N/A	N/A	3-linked β-D-galactosyl residues alternating with 4-linked 3,6-anhydro-a-L-galactose	Benzoylation	Direct scavenging of free radicals	[25,42,64]
	*Ulva pertusa*	Rha:Xyl:Glc:GlcA = 1.00:0.67:0.13:0.15	Rha:Xyl:Glc:GlcA = 1.00:0.79:0.04:0.19	β-D-Glcp A-(1→4)-α-L-Rhap3s and α-L-Idup A-(1→4)-α-L-Rhap3s	Phosphorylation		
	Blackcurrant fruits	Glc:Rha:Ara:Man:Gal:GalA = 1.00:2.31:13.29:0.95:5.13:1.96	Glc:Rha:Ara:Man:Gal:GalA = 1.00:4.35:5.65:0.23:6.65:4.35	There are pyranose rings in polysaccharides	Carboxymethylation		
	*Ulva pertusa*	Rha:Xyl:Glc:GlcA = 1.00:0.67:0.13:0.15	Rha:Xyl:Glc:GlcA = 1.00:0.79:0.04:0.19	This structure is the same as that of ref. [42] in the previous table	Phosphorylation	Regulation of antioxidant enzyme activity through the Nrf2/ARE pathway	[42,65]
	Chinese angelica	N/A	N/A	[(→4)-a-d-Glcp-(1→4)-a-d-Glcp-(1→6)-a-d-Glcp-(1→4)-a-d-Glcp-(1→4)-a-d-Glcp-(1→)]_n_	Selenization		
Anti-tumor	*E. Japonica*	N/A	N/A	→5)-linked-α-L-Araf-(1→, →4)-linked-β-D-Manp-(1→, →2,4)-linked-α-L-Rhap-(1→, →4)-linked-α-D-Xylp-(1→, →4)-linked-β-D-Galp-(1→, →2)-linked-β-D-Galp-(1→, →6)-linked-β-D-Glcp-(1→, α-D-Glcp-(4→, and t-linked-α-L-Araf	Selenization	Blocking tumor angiogenesis	[46]
	*E. Japonica*	N/A	N/A	This structure is the same as that of ref. [46] in the previous table	Selenization	Induction of apoptosis in tumor cells	[46]
	Alfalfa	Rha:Xyl:Ara:GalA:Man:Glc = 2.13:3.07:2.77:1.00:1.30:1.10	N/A	1→2, 1→4, 1→3, and 1→6 or 1→glycosidic bonds	Selenization	Unspecified	[50]
	*A. sphaerocephala*	Ara:Xyl:Man:Glc:Gal = 1.00:4.2:45.9:9.7:11.4	N/A	N/A	Sulfation	Blocking the tumor cell cycle	[70]
Anti-viral	*Sargassum ilicifolium*	N/A	N/A	N/A	Sulfation	Resists virus adsorption and invasion	[73]
	*Codonopsis pilosula*	N/A	N/A	(1→3)-linked-β-D-galactopyranosyl, (1→2,3)-linked-β-D-galactopyranosyl and (1→3)-linke-α-D-rhamnopyranosyl residues	Phosphorylation	Inhibition of virus replication	[74]
	*Chuanmingshen violaceum*	N/A	N/A	N/A	Sulfation	Activates the immune system and improves resistance to viruses	[75]
Immunomodulation	*C. paliurus*	Ara:Gal:Glc:Rha:xyl:Man:GalA:GlcA = 1.00:1.59:1.18:0.08:0.35:0.48:0.81:0.31	Ara:Gal:Glc:Rha:Man:GalA:GlcA = 1.00:1.67:1.07:0.15:0.34:1.58:0.16	N/A	Acetylation	Effect on cytokines	[27]
	*Cyclocarya paliurus*	Rha:Fuc:Ara:Xyl:Man:Glc:Gal = 0.11:0.07:3.11:0.36:0.24:0.275:3.36	Rha:Fuc:Ara:Xyl:Man:Glc:Gal = 0.27:0.07:3.51:0.25:0.17:2.41:3.32	N/A	Sulfation	Regulation of signaling pathways such as MAPK and NF-κB	[77]
	*Cyclocarya paliurus*	Rha:Fuc:Ara:Xyl:Man:Glc:Gal = 0.11:0.07:3.11:0.36:0.24:0.275:3.36	Rha:Fuc:Ara:Xyl:Man:Glc:Gal = 0.27:0.07:3.51:0.25:0.17:2.41:3.32	N/A	Sulfation	Regulation of intestinal flora	[79]
	Schisandra	N/A	Man:Glc:Gal = 1:44.8:3.71	1,4-α-D-Glcp and 1,4,6-β-D-Glcp	Carboxymethylation	Improves immune organ failure	[80]
Anti-inflammatory	*Morchella angusticeps Peck*	Ara:Man:Glc:Gal = 1.00:2.37:4.79:3.09	N/A	(1→4)-α-D-glucose, (1→6)-α-D-galactose, (1→2)-α-D-mannose, and (1→5)-α-D-arabinose; and the branches were found to be (→2→6)-α-D-mannose, (1→2→6)-α-Dglucose, and (1→2→6)-β-D-galactose	Acetylation	Inhibition of NF-κB and MAPK signaling pathways	[26]
	*Pholiota nameko*	Man:Glc:Gal:Ara:Rha = 6.4:38.6:27.1:20.5:7.4	Man:Glc:Gal:Ara:Rha = 7.3:44.9:23.6:15.7:8.5	The main chains were1,4-linked Glcp, 1,6-linked Gal*p*, 1,2- linked Rha*p*, and 1.6-linked Man*p* with terminals of t-linked Glc*p*, t-linked Ara*f* The side chains change from 1,4,6-linked Gal*p*, 1,2,5-linked Ara*f* to 1,4,6-linked Gal*p*	Phosphorylation	Inhibition of PI3K/AKT signaling pathway	[82]
	*Morchella angusticeps Peck*	Ara:Man:Glc:Gal = 1.00:2.37:4.79:3.09	N/A	Its structure is the same as that of ref. [26] in the previous table	Acetylation	Inhibition of NO and PGE2 production	[26]
	*Codium fragile*	N/A	N/A	N/A	Sulfation	Affects cytokine secretion	[83]

### 3.6. Other Biological Activities

Many pharmacological experiments have shown that both sulfated polysaccharides naturally present in plants and those obtained via chemical modification have good anticoagulant effects [84,85]. Through testing routine coagulation indicators (APTT, TT, PT), it was found that the products modified by phosphorylation and carboxymethylation have significant anticoagulant activity compared to original polysaccharides [86]. This indicates that in addition to the well-known sulfated polysaccharides, the introduction of other anionic groups into polysaccharides increases their anticoagulant activity, which can greatly enrich the types of anticoagulants and provide a reference in the search for naturally active anticoagulants. In addition to this, chemically modified polysaccharides also have liver-protective effects. Selenizing polysaccharides (sCAPs) from Chinese angelica can significantly reduce the levels of ALP, ALT and AST in the serum of liver-injury mice. Moreover, sCAP significantly alleviates pathological changes in the liver while also inhibiting the expression of p-ERK, indicating that selenization modification can enhance the hepatoprotective effect of sCAP [65]. Sulfated (SLEP) and carboxymethylated (CLEP) extracellular polysaccharides from *Lachnum* YM240 can both have a hypolipidemic effect, but the hypolipidemic effect of CLEP is more significant [87].

Moreover, the potential of marine polysaccharides should not be overlooked. Marine polysaccharides have attracted much attention because of their abundant sources, special molecular structures and extensive biological activities [88]. The biological activities of marine-derived polysaccharides and their derivatives have proved to be anti-thrombotic, antitumor, antioxidative, immunomodulatory, etc., which have wide application prospects in functional foods, drugs and other fields [6,29,33,89].

## 4. Factors Affecting the Bioactivity of Chemically Modified Polysaccharides

### 4.1. Introduction of Different Chemical Modification Groups

As is well known, the structure of substances determines their function, and when the structure of polysaccharides changes, their function tends to change as well. For example, when polysaccharides WPMP-1 and WPMP-2 were extracted from Polygonum multiflorum, both the methylation and NMR results indicated that the main chain structure of WPMP-1 was composed of 1, 4-Glcp. In contrast, the main chain of WPMP-2 is 1, 3, 5-Araf and 1, 2, 4-Rhap. Therefore, the significant differences in structure also lead to functional differences between the two purified polysaccharides, such as WPMP-2, showing stronger immune regulatory activity than WPMP-1 [90].

Recently, a study using ^13^C NMR technology has characterized the structure of onion polysaccharide. At the same time, phosphate and acetyl groups were introduced in onion polysaccharides, and antioxidant analysis was conducted. The results of in vitro antioxidant activity showed that the antioxidant effect of phosphated onion polysaccharide was the strongest compared to original and acetylated polysaccharides, which approached the activity of vitamin C [91]. In addition, acetylated (AcP) and carboxymethylated polysaccharides (CM-Ps) from bitter gourd were successfully prepared by introducing acetyl and carboxymethyl groups on the main chain of the polysaccharide using bitter gourd polysaccharide (P) as the raw material. Antioxidant tests were conducted on the three polysaccharides, with the results showing that CM-Ps had the strongest antioxidant effect. It was speculated that this may be because, with the introduction of carboxymethyl groups, the negative charge on the surface of polysaccharide increases, resulting in the construction of negatively charged hydrophilic surface structures that increase their water solubility and thus enhance their antioxidant activity [9]. However, the same chemical modifications applied to different polysaccharides often exhibit vastly different activities, which may be closely related to the unique structure of the polysaccharides themselves. Therefore, it is necessary to select appropriate chemical modification methods based on the structure of polysaccharides themselves during chemical modification [92,93].

### 4.2. DS and Substituent Position

The DS (degree of substitution) is an important indicator for evaluating the success of chemically modified polysaccharides. However, even if polysaccharides with different DS values are prepared using the same chemical modification method, their activity often varies greatly. Generally, it is believed that the biological activity of modified polysaccharides is directly proportional to the size of the DS values, i.e., the larger the DS values are, the greater the activity is. For example, different DS carboxymethylated blackcurrant fruit polysaccharides (CRNPs) were prepared from blackcurrant fruits using the carboxymethylation method. On the other hand, the in vitro antioxidant results indicated that the antioxidant activity of CRNPs is enhanced compared to unmodified polysaccharides. Under the test conditions of the in vitro hemolytic protective effect on red blood cells, the activity of CRNPs also increases with the increase in the DS [64]. In addition, arabinoxylans is extracted from the seed shell of *Plantago*, and sulfate is added to obtain products with different DS values. After in vitro anti-HSV-1 activity testing, it was found that the polysaccharide with the highest DS had the strongest anti-HSV-1 activity [94]. However, most experiments show that the relationship between DS values and biological activity does not seem to be so simple. Briefly, the fruiting bodies of polysaccharides from *Russula virescens* (SRVP) with DS values between 0.34 and 0.73 were prepared, and the in vitro activity tests showed that SRVP1-20 with the DS of 0.68 had the strongest antibacterial and antitumor activity [15]. At the same time, other results can prove this viewpoint as well. Researchers have used *Sagittaria trifolia* as a raw material to prepare three sulfated polysaccharides with different DS values and evaluated their antioxidant activity. The results showed that the antioxidant activity decreased with the increase in the DS value [95].

In addition to the DS value, the biological activity of chemically modified polysaccharides is also closely related to the sites of the substituent introduction. Some scholars modified carrageenans with sulfation via selective sulfation. A total of eleven samples were prepared. Sample 8 has the same DS as sample 11, but sample 8 is replaced by a sulfuric acid group at C4, and sample 11 is replaced by a sulfuric acid group at C2. The results obtained from samples 8 and 11 can indicate that substitution by sulfate groups at C4 appears to have better anticoagulant activity than substitution at C2 [96].

### 4.3. Monosaccharide Molar Ratio and Glycosidic Bond Link Order

**Figure 8:**
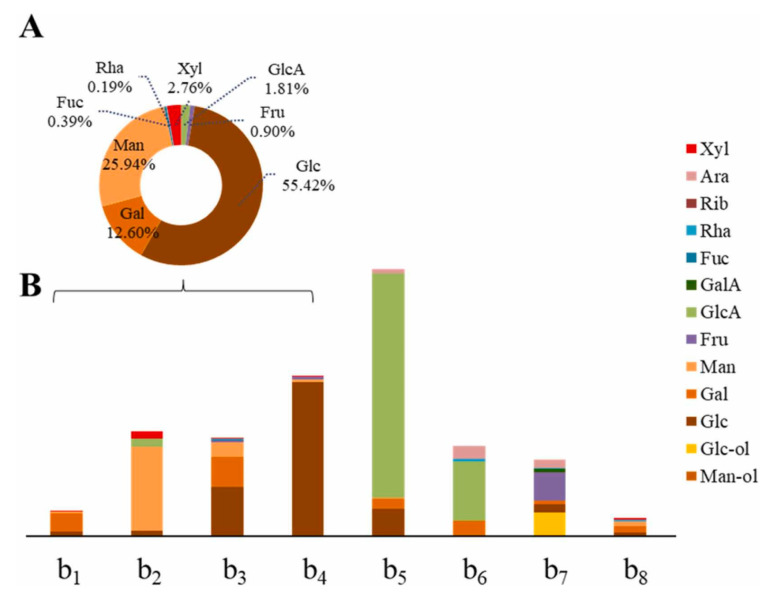
The polysaccharide components were analyzed using the RPLC–ESI–MRM–MS method. (**A**) Composition and proportion of monosaccharides in all four fungi polysaccharides. (**B**) Analysis of monosaccharide composition and content of individual polysaccharides from edible plants and fungi. **b1**: Ganoderma lucidum polysaccharide; **b2**: Auricularia auricular-judae polysaccharide; **b3**: Armillaria mellea polysaccharide; **b4**: Hericium erinaceus polysaccharide; **b5**: Panax ginseng polysaccharide; **b6**: Aralia elata bud polysaccharide; **b7**: Platycodon grandiflorum polysaccharide; **b8**: Stigma maydis polysaccharide. This figure was adapted from Gao et al. [97].

Monosaccharide composition analysis is the basis for studying the structural properties and the structure–activity relationships of polysaccharides. Common analytical methods for determining monosaccharide composition currently include liquid chromatography [10], liquid chromatography–mass spectrometry (Figure 8) [97], gas chromatography [64] and gas chromatography–mass spectrometry [41]. A large number of studies have concluded that the chemical modification of natural polysaccharides generally leads to changes in the molar percentage of monosaccharide composition, but it does not lead to changes in monosaccharide composition [42,64,77,82]. Natural polysaccharide from *Undaria pinnatifida* (UPPS-B1) and its sulfated derivative (S-UPPS-B1) were used for the determination of monosaccharide composition by GC–MS. The molar ratio of monosaccharides in UPPS-B1 was Glc, Man, Xyl and Gal at 12.0:8.7:7.9:9.8. After sulfation modification, the proportion of Xyl, Glc and Gal were significantly decreased, and the antitumor activity of sulfated polysaccharides was enhanced [98]. However, in rare cases, the chemical modification of polysaccharides can lead to changes in the composition of monosaccharides. For example, monosaccharides in the polysaccharide (CP) from *Cyclocarya paliurus* were Ara, Gal, Glc, Rha, xyl, Man, GalA and GlcA. Surprisingly, the monosaccharide composition of the acetylated polysaccharide (AC-CP) from *Cyclocarya paliurus* was Ara, Gal, Glc, Rha, Man, GalA and GlcA, without Xyl composition. At the same time, comparing immunoregulatory activity, AC-CP activity is superior to CP [27].

Additionally, chemical modifications can also lead to a certain degree of alteration of glycosidic bond linkages. Phosphorylated polysaccharide (PPN) from *Pholiota nameko* was prepared via chemical modification of polysaccharide (SPN) from *Pholiota nameko.* The methylation results showed that there was no change in the main chain structure of SPN and PPN, but the side chains of SPN underwent phosphorylation, transitioning from 1, 2, 5-Ara*f* to 1, 2, 4-Glc*p* linkage. At the same time, the ability of PPN to scavenge free radicals and its anti-inflammatory effect against LPS-induced RAW 264.7 cells are stronger than those of SPN [82]. Therefore, the chemical modification of polysaccharides will lead to changes in monosaccharide composition, molar ratio, glycoside linkage sites and other structures, further leading to changes in biological activity. However, so far, there is no exact evidence to show the relationship between polysaccharide structure and activity, which is still the focus of a future research direction.

### 4.4. Molecular Weight (M_W_)

At present, the most common method for the determination of molecular weight in polysaccharides is high-performance gel permeation chromatography (HPGPC), which is widely used in the analysis and preparation of polysaccharides due to the advantages of simple operation and short time [85]. The molecular weights *of Enteromorpha prolifera* polysaccharide (PEP) and its enzymatic degradation products (LEP) were 147.8 KDa and 44.8 KDa, respectively. By sulfating PEP and LEP, the products were SPEP (176.3 KDa) and SLEP (59.9 KDa), respectively. The molecular weight of polysaccharides increased after sulfated modification. In addition, the antioxidant activities of PEP, LEP, SPEP and SLEP were measured and compared. The results showed that SLEP had the strongest antioxidant activity due to low Mw and high sulfate-group content. Therefore, the molecular weight and sulfate groups have obvious effects on the antioxidant activity of *E. prolifera* polysaccharide [99]. However, in contrast to the previous example, the molecular weight of polysaccharide (SGP) from *Sphallerocarpus gracilis* is 743 KDa, and S-SGP is prepared by sulfation using the CSA/Pyr method with SGP as the raw material. The results indicate that the molecular weight of S-SGP is significantly reduced to 212 KDa. In spite of this, the antioxidant activity of the sulfated modification product S-SGP was instead elevated [100]. According to the current evidence, the biological activity of modified polysaccharides is related to molecular weight to some extent, but the chemical modification of polysaccharides often leads to changes in the monosaccharide molar ratio, Mw, degree of substitution, glucoside linkage and other factors. Therefore, there is no direct evidence to show the exact relationship between molecular weight and biological activity, and the structure–activity relationship still needs to be further studied.

## 5. Conclusions and Prospects

At present, the application of chemically modified polysaccharides has been implemented in the biomedical and food industries, along with the cosmetics and materials industries and antibacterial agent research and development. Therefore, it has high practical and economic value. However, due to the large molecular weight and complex advanced structure of polysaccharides, it is also a great challenge to study the structure–activity relationship of polysaccharides. Changes in the activity of chemically modified polysaccharides are often related to the type, position and quantity of substituent groups (expressed by DS or content), monosaccharide composition and molecular weight, and the sequence of glucoside linkage [93,100,101]. This unpredictability in the structure–activity relationship also leads to the biological activity of some chemical modifications being less than expected and even to the phenomenon of reduced activity [91,102].

Therefore, polysaccharide modification should not overlook decoration through various methods; instead, it should be according to their own unique structure modification. Despite the promising prospects of chemically modified polysaccharides in recent years, the following challenges remain: (1) homogeneous purified polysaccharides are rarely used in polysaccharide modification. A low purity of lead compounds will lead to an unstable DS and substitution sites after chemical modification and poor experimental reproducibility, and it will increase the difficulty of studying the structure–activity relationship. (2) At the same time, the controllability of the reaction is not high, and the DS and molecular weight of different batches of modified products under the same reaction conditions are inconsistent, making products difficult to control. Therefore, increasing the controllability of the DS and molecular weight of the modified products is very important in the study of the structure–activity relationship of modified polysaccharide products. (3) Catalysts used in many chemical modification methods have a strong toxicity and too many by-products that are difficult to separate, resulting in a low yield of target products. (4) At present, the structure–activity relationship of chemically modified polysaccharides is still relatively shallow, and the mechanism research is not clear, so it is necessary to deepen the study of the structure–activity relationship. However, with the continuous development of scientific research equipment and the gradual development of analytical methods, these problems will be solved, and the chemical modification of polysaccharide methods will be gradually improved so that polysaccharides and their modified products will have a wider range of applicability.

## Figures and Tables

**Table 1:** The advantages and disadvantages of several common methods for polysaccharide chemical modification.

Modification	Modification Method	Reagents	Advantages	Disadvantages	**References**
Acetylation	Acetic anhydride (acetic acid) method	Acetic anhydride (or acetic acid), pyridine (or 4-DMAP), formamide	Simple operation steps and short response time	Pyridine is highly irritating and neurotoxic; 4-DMAP is expensive and difficult to be exploited on a large scale	[24,25]
Sulfation	Sulfamic acid method	Sulfamic acid, N, N-dimethylformamide	Mild reactions and low toxicity	Lower product DS and more side effects	[33,34]
	Sulfur trioxide-pyridine method	Sulfur trioxide, pyridine, formamide	Simple operation and high product DS	Sulfur trioxide is more expensive and only suitable for small-scale production	[36]
	Concentrated sulfuric acid method	Concentrated sulfuric acid (CSA), n-butanol, ammonium sulfate	The reaction is stable, less toxic and less costly	CSA is too acidic, which can easily cause polysaccharide carbonization and sugar chain degradation	[37]
	Chlorosulfate-pyridine method	Chlorosulfonic acid, pyridine, formamide	Easy operation, high product yield, high DS	Chlorosulfonic acid is unstable and acutely toxic	[38]
Phosphorylation	Acid and Anhydride Method	Phosphoric acid (phosphoric anhydride), DMSO	Simple operation, low equipment requirements	The exothermic reaction is prone to polysaccharide degradation	[40]
	Phosphorous oxychloride	POCl_3_	Rapid reaction time, simple operation, high DS	More toxic by-products, irritating gases from the reaction	[16,41]
	Phosphate method	Sodium tripolyphosphate (STPP), Sodium trimetaphosphate (STMP)	Easy to operate and less prone to polysaccharide degradation	Low reaction activity, low DS and yield	[42,43]
	Phosphorus pentoxide method	Methanesulfonic acid, P_2_O_5_	Short reaction time	P2O5 is more acidic and prone to polysaccharide degradation	[44]
Selenization	Selenate method	Nitric acid (or glacial acetic acid), sodium selenite	Lower cost	Long reaction time and complex steps	[50]
	Selenium Oxychloride Method	Selenium Oxychloride (SeOCl_2_)	Simple operation steps	SeOCl_2_ is easily decomposed, and the reaction will produce irritating and toxic gases	[51]

## Data Availability

Not applicable.
